# Allergic contact dermatitis to toluene-sulfonamide-formaldehyde resin: still relevant?^☆^

**DOI:** 10.1016/j.abd.2023.04.009

**Published:** 2024-02-20

**Authors:** Rosana Lazzarini, Bruna Barravieira Masselli, Mariana de Figueiredo da Silva Hafner

**Affiliations:** Dermatology Clinic, Santa Casa de São Paulo, Irmandade da Santa Casa de Misericórdia de São Paulo, São Paulo, SP, Brazil

Dear Editor,

Toluene-sulfonamide-formaldehyde resin (TSFR), used in nail polishes since 1939, aims at promoting durability, adhesion, and increased shine. However, it represents a potent allergen, as its chemical structure does not change with the modification in physical state; therefore, TSFR in dry nail polish can continue to sensitize locally, in addition to producing ectopic allergic contact dermatitis (ACD), mainly on the face (eyelids, perioral area, and chin) and on the cervical region. In previous decades, TSFR proved to be frequent and relevant in patch tests around the world. Data from 2001 to 2016 from the North American Contact Dermatitis Group (NACDG) revealed that 2% of the 38,775 patients who underwent patch testing during that period resulted from allergic or irritant contact dermatitis to products used on nails. Among these cases, 273/755 (36.2%) were due to TSFR.^1^

In Brazil, although TSFR is not among the 30 substances in the standard series, it is present in the complementary cosmetic battery, used in our country for about 20 years. Furthermore, TSFR is also listed as one of the allergens in the latest expanded Brazilian battery.

National studies have shown TSFR positivity frequencies of 14% to 29% among all patch tests carried out concomitantly with the standard battery and the cosmetic one.^2^ This fact could be justified by the large consumption of nail polish in the Brazilian market, which ranks second in the world in this category, behind only the United States, according to the National Health Surveillance Agency (ANVISA, *Agência Nacional de Vigilância Sanitária*).^3^ Research shows that around 27% of Brazilian women change the color of their nail polish at least once a week.^3^

However, studies from other countries have shown that the frequency of sensitization by TSFR has decreased in recent years, and, at the same time, other substances present in nail polish have become common allergens. The present study was carried out to demonstrate the relevance of this fact in our country, with retrospective research of data related to positive patch tests for TSFR between January 2011 and December 2022, which were collected from the spreadsheets used in the service. The results were submitted to statistical analysis using the Mantel-Haenszel chi-square test (to demonstrate whether there was a linear downward trend in the obtained values).

During the period, 1,280 patch tests were carried out, of which 681 were tested for TSFR (53.2%) due to suspected ACD caused by cosmetics. To carry out the tests, until 2021, the standard Brazilian battery associated with the complementary series of cosmetics was used, and, as of 2022, the expanded Brazilian battery (the latter used in all patients submitted to contact tests in the service during the year).

Among the patients in the group tested for TSFR, 65 (9.5%) tested positive. Of these, 58 (89.2%) were relevant at the time of the patch test and seven (10.8%) were previously relevant. However, when analyzing the data in Table 1 and Fig. 1, a decreasing number of positive tests for this substance is observed over the years. The analysis of the results showed a significant change in positive reactions in the analyzed years, with a linear trend of decreasing proportions of the studied percentages (p < 0.0001).

**Table 1:** Distribution of patch tests for TSFR^a^ between 2011 and 2022.

Year	Patients tested for TSFR (n)	Positive reactions (n/%)	Current relevant reactions (n/%)	Reactions with previous relevance (n/%)
2011	42	16 (38)	16 (38)	0 (0)
2012	32	5 (15.6)	5 (100)	0 (0)
2013	25	10 (40.0)	10 (100)	0 (0)
2014	74	11 (14.9)	11 (100)	0 (0)
2015	92	6 (6.5)	5 (83.4)	1 (20)
2016	43	4 (9.3)	3 (75)	1 (33.4)
2017	40	2 (5.0)	2 (100)	0 (0)
2018	17	2 (11.8)	2 (100)	0 (0)
2019	52	2 (3.8)	1 (50)	1 (50)
2020	30	1 (3.4)	0 (0)	1 (100)
2021	94	3 (3.2)	0 (0)	3 (100)
2022	140	3 (2.1)	3 (2.1)	0 (0)
Total	681	65 (9.5)	58 (89.2)	7 (10.8)

Mantel-Haenszel chi-square test, p < 0.0001.

a TSFR, Toluene-sulfonamide-formaldehyde resin.

**Figure 1:**
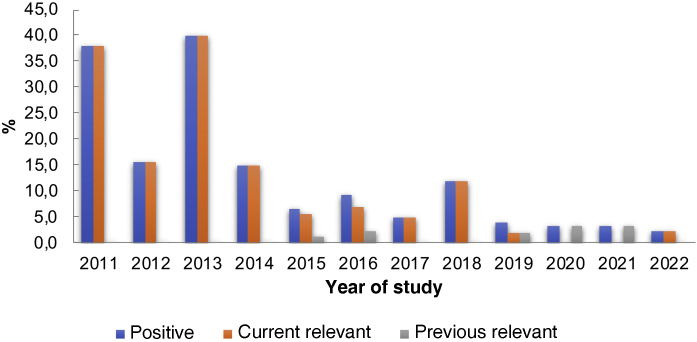
Decrease in frequency of positive and relevant tests for TSFR between 2011 and 2022.

This fact could be attributed to some hypotheses: 1) Greater knowledge of the characteristics of ACD by nail polish by physicians and the lay population, which would lead to a rapid “diagnosis” and, therefore, cure of the dermatitis, without performing patch tests; 2) Greater variety of nail polishes without TSFR available on the market, as observed by the authors with the practice of checking the labels of products brought in by the patients.

A similar study carried out in Australia in 2018 disclosed a decreasing frequency of positive tests for TSFR but with lower percentages than those found in our country. It is evident this allergen is still relevant in Brazil, even with the decrease in frequency in recent years, probably due to the cultural habit of applying nail polish weekly. The incorporation of TSFR into the expanded Brazilian series, which should be used in all patients undergoing patch testing, will allow knowing the actual proportion of its sensitization frequency in the general population.^4^

Although contact dermatitis that occurs on the facial and cervical regions is classically associated with nail polish, there are other differential diagnoses. A retrospective study carried out on contact dermatitis on the eyelids revealed other allergens, such as methylisothiazolinone and fragrances, present in various cosmetics (shampoos, for instance). Clinically, ACD lesions caused by nail polish are commonly unilateral, located on the eyelids, chin, and cervical region, while those caused by shampoo and cosmetics applied directly to the face are bilateral and symmetrical. Anyway, the diagnosis is not always obvious, requiring confirmation by patch tests.^5^

Moreover, other potential sensitizers present in nail polish can cause ACD, such as phthalates, acrylates and nitrocellulose, which also reinforces the need for adequate investigation of the cases.

Another issue to be addressed is product labeling, which does not always follow standardization. A Brazilian publication showed that many so-called “hypoallergenic” nail polishes contained potentially allergenic substances in their composition.^6^ Another American study from 2018 showed that there was no uniformity in the labels of hypoallergenic nail polishes among the assessed brands.^7^

The present study, despite showing results from a single center, suggests a downward trend in the frequency of positive tests for TSFR in the last ten years (from 16% to 2.1%), although such percentages are still high compared to those from other countries. Therefore, further studies are necessary to determine the actual importance of TSFR as an allergen to be added to the standard series or remain part of a complementary one.

## Financial support

None declared.

## Authors’ contributions

Rosana Lazzarini: Study planning, analysis, and interpretation of data; intellectual participation in the propaedeutic and/or therapeutic conduct of the cases; critical review of content; approval of the final version of the manuscript.

Bruna Barravieira Masselli: Data collection; critical review of the content; approval of the final version of the manuscript.

Mariana de Figueiredo da Silva Hafner: Data analysis and interpretation; intellectual participation in the propaedeutic and/or therapeutic conduct of the cases; critical review of content; approval of the final version of the manuscript.

## Conflicts of interest

None declared.
